# Common signatures of neutrophils in diverse disease conditions

**DOI:** 10.1038/s41421-025-00818-9

**Published:** 2025-08-01

**Authors:** Di Wu, Ying Cao, Hongjie Chen, Minghao Du, Tingting Wang, Jiarui Zhao, Mengyuan Li, Wenjing Wu, Huixuan Zhang, Ence Yang, Jing Yang, Jian Chen

**Affiliations:** 1 School of Basic Medical Sciences, Capital Medical University & Chinese Institute for Brain Research, Beijing, China; 2 Beijing Institute for Brain Research, Chinese Academy of Medical Sciences & Peking Union Medical College, Beijing, China; 3 Academy for Advanced Interdisciplinary Studies, Peking University, Beijing, China; 4 Department of Medical Bioinformatics, School of Basic Medical Sciences, Peking University Health Science Center, Beijing, China; 5 Changping Laboratory, Beijing, China; 6 State Key Laboratory of Membrane Biology, School of Life Sciences, Peking University, Beijing, China; 7 IDG/McGovern Institute for Brain Research, Peking University, Beijing, China; 8 Peking University Third Hospital Cancer Center, Beijing, China; 9 Peking Union Medical College Hospital, Beijing, China

**Keywords:** Immunology, Cancer

Dear Editor,

Neutrophils are traditionally recognized for their pro-inflammatory roles. However, accumulating evidence has begun to highlight the plasticity of transcriptional states of neutrophils during pathological insults. Whether such unconventional neutrophils share commonality across diverse disease conditions is incompletely understood. Here, we systematically profile over 500,000 neutrophils in key immune compartments and disease-inflicted tissues of the mouse models of metabolic disorder, autoimmunity, tissue damage, peripheral cancers, and intracranial gliomas. Of importance, we observe two distinct neutrophil clusters with unique immune features. The first cluster, characterized by Cd274, Vegfa, and antigen presentation, is highly enriched within the diseased tissues. In contrast, the second cluster with elevated Cd244 and type 2 immune response but reduced maturation markers primarily emerges in the peripheral blood and spleen of specific disease scenarios. These results have elucidated the common signatures of neutrophils in response to various pathological conditions, providing a valuable resource for the research of neutrophil biology.

Neutrophils are the most abundant immune cells in the body and can account for > 50% of white blood cells. It has long been documented that neutrophils respond to various pathological insults by phagocytosis, releasing pro-inflammatory factors (i.e., degranulation), or forming neutrophil extracellular traps (i.e., NETosis)^1,2^. Notably, recent studies of single-cell RNA sequencing (scRNA-seq) by colleagues and us have begun to define unconventional transcriptional states of neutrophils in different cancers, e.g., liver cancer, lung cancer, breast cancer, pancreatic cancer, and glioma^3–8^. However, whether neutrophil transcriptional states share commonality across diverse disease conditions is incompletely understood.

We systematically profiled neutrophils in the mouse disease models of metabolic disorder (high-fat diet, HFD), autoimmunity (experimental autoimmune encephalomyelitis, EAE), tissue damage (acute lung injury, ALI), peripheral cancers (Lewis lung carcinoma (LLC), B16 melanoma, and MC38 colorectal carcinoma), and intracranial tumors (LCPNS or LCPNS-SIIN gliomas^8^). Neutrophils were isolated by fluorescence-activated cell sorting (FACS) from the key immune compartments, i.e., bone marrow, spleen, and peripheral blood, and the diseased tissues, i.e., liver, spinal cord, lung, or tumors, and pooled for scRNA-seq profiling (Supplementary Table S1). After the quality control, a total of 534,689 neutrophils were obtained in the scRNA-seq dataset (Fig. 1a). Notably, neutrophil populations in different tissues or disease conditions could be integrated (Fig. 1a, e, f; Supplementary Fig. S2), supporting a successful correction of batch effects. Among the defined transcriptional states, the progenitor cell marker *Cd34*, the immature neutrophil marker *Camp*, and the proliferation marker *Mki67* decreased as granulocyte-monocyte progenitors (GMPs) differentiated into more mature populations (Fig. 1b; Supplementary Fig. S1a). Meanwhile, an overall increasing trend of classic neutrophil markers *Cxcr2* and *S100a8* was observed with neutrophil maturation (Fig. 1b). This putative differentiation path could be visualized by the RNA velocity (Fig. 1c) and the pseudotime trajectory analyses (Fig. 1d), and those neutrophil transcriptional states had the distinct enrichment of the targeted genes of specific transcription factors (Supplementary Fig. S1b). Neutrophil transcriptional states profoundly differed in the diseased tissues (Fig. 1e; Supplementary Fig. S2a), peripheral blood (Fig. 1f), and spleen (Supplementary Fig. S2b). Neutrophils in the bone marrow were also affected, albeit to a lesser extent, by disease conditions (Supplementary Fig. S2c), e.g., there was a significant decrease of Neu_Cd101 in the ALI and LLC models.

**Fig. 1:**
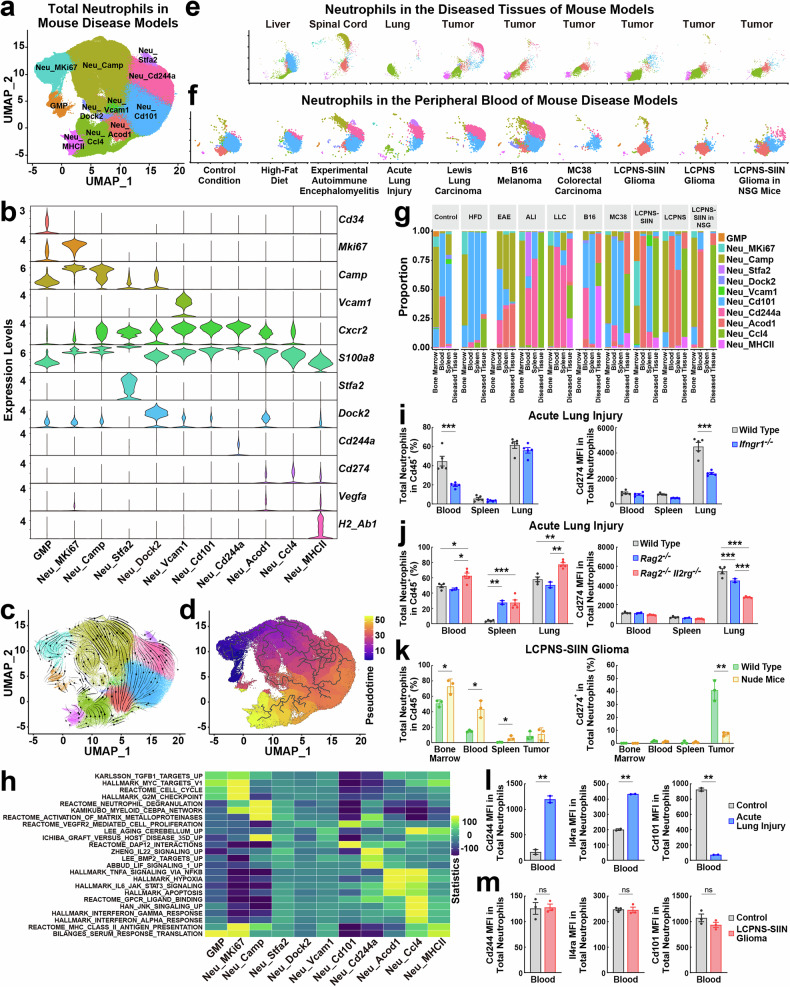
Common signatures of neutrophils in different disease conditions. **a** UMAP plot of neutrophil transcriptional states in the pooled scRNA-seq dataset of the bone marrow, peripheral blood, spleen, and disease-inflicted tissues of different mouse models. GMP, granulocyte-monocyte progenitor. **b** Violin plots of the signature genes for neutrophil transcriptional states. **c** RNA velocity plot of neutrophil transcriptional states. **d** Pseudotime trajectory analysis of neutrophil transcriptional states. **e**, **f** UMAP plots of neutrophils in the diseased tissues (**e**) or the peripheral blood (**f**) of different mouse models. **g** Proportions of neutrophil transcriptional states in the bone marrow, peripheral blood, spleen, and diseased tissues of different mouse models. **h** Enrichment score heatmap of the gene sets of cytokine-related pathways in neutrophil transcriptional states. **i**, **j** C57BL/6 wild-type or *Ifngr1*^*–/–*^ mice (**i**) or *Rag2*^*–/–*^ and *Rag2* ^*–/–*^ *Il2rg*^*–/–*^ mice (**j**) were subjected to ALI. Total neutrophils in the peripheral blood, spleen, and lung tissues and their mean fluorescence intensity (MFI) of Cd274 expression were assessed by FACS. Data are shown as mean ± SEM; *P < 0.05, **P < 0.01, ***P < 0.001 (ANOVA test). **k** BALB/c wild-type or nude mice were utilized in the LCPNS-SIIN glioma model. Total neutrophils and Cd274^+^ neutrophils in the bone marrow, peripheral blood, spleen, and tumors were examined by FACS. Data are shown as mean ± SD; *P < 0.05, **P < 0.01 (ANOVA test). **l**, **m** C57BL/6 wild-type mice were subjected to ALI (**l**) or LCPNS-SIIN gliomas (**m**). MFI of Cd244, Il4ra, or Cd101 expressed by neutrophils in the peripheral blood was quantified by FACS. Data are shown as mean ± SD; ns not significant, **P < 0.01 (Student’s *t-*test).

In this comprehensive profiling of neutrophils across disease conditions, we observed two neutrophil clusters with unique immune characteristics. The first cluster included Neu_Acod1, Neu_Ccl4, and Neu_MHCII, which expressed the immunosuppressive gene *Cd274* (also known as Pd-l1; Fig. 1b) and myeloid recruitment-related chemokines *Ccl3* and *Ccl4* (Supplementary Fig. S1a). Additionally, Neu_Acod1 and Neu_MHCII enriched the angiogenic factor *Vegfa*, and Neu_MHCII expressed the MHC class II genes such as *H2-Eb1* (Fig. 1b; Supplementary Fig. S1a). In line with the previous studies identifying Cd274^+^ neutrophils in the immunosuppressive tumor microenvironment of different cancers^4,7,8^, we detected Neu_Ccl4 in all the examined peripheral or intracranial tumors (Fig. 1e, g). Consistent with the recent reports on the critical function of Vegfa^+^ or MHC-II^+^ neutrophils in antitumor immunity^5,9,10^, Neu_MHCII was present in the peripheral or intracranial tumors except those in the immunodeficient NSG mice (Fig. 1e, g). Moreover, Neu_Ccl4 was present in the livers of HFD-fed mice and the lungs of ALI (Fig. 1e) while being almost absent in the peripheral blood (Fig. 1f), spleen (Supplementary Fig. S2b), and bone marrow (Supplementary Fig. S2c) of all the disease models. FACS analyses confirmed the presence of both Neu_Ccl4 as Cd274^+^ MHCII^low^ and Neu_MHCII as Cd274^+^ MHCII^high^ neutrophils in the LLC tumors, while only Cd274^+^ MHCII^low^ neutrophils were in the lungs of ALI (Supplementary Fig. S6a).

The Over Representation Analysis (ORA) of the gene sets of signaling pathways revealed that Neu_Ccl4 might be regulated by specific cytokines, e.g., tumor necrosis factor (TNF) or interferons (IFNs), as well as by hypoxia (Fig. 1h; Supplementary Fig. S3). Meanwhile, Neu_MHCII was related to the pathway of antigen presentation, as expected (Fig. 1h; Supplementary Fig. S3). In the published scRNA-seq dataset (GSE202186) of mouse immune cells exposed to various cytokines^11^, Neu_Ccl4 was also associated with the TNF and IFN signals (Supplementary Fig. S4). Of importance, the in vitro treatment of conventional neutrophils from mouse bone marrow with TNF-α, IFN-α, IFN-β, or IFN-γ was sufficient to elevate their expression of Cd274, as assessed by FACS (Supplementary Fig. S5a). Bulk RNA-seq analyses further validated that those cytokines effectively induced the top 100 signature genes of scRNA-seq-defined Neu_Ccl4 (Supplementary Fig. S5b). On the contrary, transforming growth factor beta (TGF-β) produced a minor effect, while interleukin 6 (IL-6) entirely failed to induce the Neu_Ccl4 phenotype (Supplementary Fig. S5a, b). In addition, genetic deletion of the IFN-γ receptor 1 (*Ifngr1*) blocked the in vitro induction of Neu_Ccl4 by IFN-γ but not other cytokines (Supplementary Fig. S5c). We further examined the in vivo involvement of IFN signals in eliciting Neu_Ccl4. Cd274^+^ neutrophils were robustly detected by FACS in the lungs of wild-type mice in the ALI model (Supplementary Fig. S6c), but their appearance was diminished in the *Ifngr1*^*–/–*^ mice (Fig. 1i). Moreover, Cd274^+^ neutrophils became reduced in the lungs of *Rag2*^*–/–*^ or *Rag2*^*–/–*^ *Il2rg*^*–/–*^ mice subjected to ALI (Fig. 1j). Similarly, Cd274^+^ neutrophils were present in the tumors but not the peripheral blood or spleen of wild-type mice in the LCPNS-SIIN glioma model (Supplementary Fig. S6d), however, their presence was abolished in the nude mice that severely lacked T and B lymphocytes (Fig. 1k). Together, these in vitro and in vivo results suggested the involvement of lymphocyte-derived IFNs in the Neu_Ccl4 induction.

The second unique cluster of neutrophils, Neu_Cd244a, was characterized by *Cd244*, contributing to immunosuppression, and *Il4ra*, a key component in type 2 immune response (Fig. 1b; Supplementary Fig. S1a). In contrast to the predominant presence of Neu_Ccl4 within diseased tissues, Neu_Cd244a was more enriched in the peripheral blood and spleen of specific disease conditions, e.g., ALI or LLC, but not other models, e.g., MC38 colorectal carcinoma, LCPNS glioma, or LCPNS-SIIN glioma (Fig. 1f, g; Supplementary Fig. S2b). FACS validated the presence of Neu_Cd244a in the peripheral blood of the ALI or LLC model, showing their increased expression of Cd244 and Il4ra but decreased expression of maturation marker Cd101 (Fig. 1l; Supplementary Fig. S6b). On the contrary, there was no significant change in Cd244, Il4ra, or Cd101 expressed by neutrophils in the peripheral blood of the LCPNS-SIIN glioma model (Fig. 1m), consistent with the absence of scRNA-seq-defined Neu_Cd244a in this disease scenario (Fig. 1f). We verified the correlation between mRNA expression and protein levels of Cd244 and Cd274 in neutrophils. Cd244^+^ neutrophils in the peripheral blood and Cd274^+^ neutrophils in the lungs of the ALI model were FACS-sorted based on their protein expression, and bulk RNA-seq analyses showed the distinct enrichment of *Cd244* and *Cd274* mRNAs in the two populations, respectively (Supplementary Fig. S6e). To examine the potential immunosuppressive function of Neu_Cd244a, FACS-sorted Cd244^+^ neutrophils were co-cultured with OVA_257–264_ peptide-activated OT-1 Cd8^+^ T cells. As a positive control of this in vitro assay, Cd274^+^ neutrophils triggered the upregulation of Tim3 and Lag3, the two key markers for Cd8^+^ T cell exhaustion^12^, while Pd1 levels were unaffected (Supplementary Fig. S7a). Cd244^+^ neutrophils effectively elevated the expression of Tim3 and Lag3 in Cd8^+^ T cells (Supplementary Fig. S7a). Moreover, administration of an anti-Cd244 neutralizing antibody significantly boosted the expression of a collection of pro-inflammatory cytokines and chemokines in the lungs of the ALI model (Supplementary Fig. S7b), although the neutrophil-specific Cd244 signal in this disease context remained to be characterized by future research.

In sum, this study has profiled neutrophil transcriptional states and elucidated their common features in different mouse disease models, providing a valuable resource for investigating the pleiotropic functions of neutrophils in pathophysiological contexts. Furthermore, the identification of two distinct neutrophil clusters has implicated novel entry points for diagnostic strategies to stratify patients with specific disease conditions.

## Supplementary information

Supplementary information

## Data Availability

The sequencing data generated in this study are publicly available without restriction. scRNA-seq and bulk RNA-seq FASTQ files have been deposited in the Genome Sequence Archive at the National Genomics Data Center under the accession number CRA020963 (https://ngdc.cncb.ac.cn/gsa/browse/CRA020963). The fully processed single-cell expression matrix, together with cell-level metadata, UMAP coordinates, and gene annotations, is provided as an h5ad file (Scanpy-compatible) from Figshare (10.6084/m9.figshare.29538791).

## References

[1] Mayadas, T. N., Cullere, X. & Lowell, C. A. *Annu. Rev. Pathol.* **9**, 181–218 (2014). 10.1146/annurev-pathol-020712-164023 PMC4277181 24050624

[2] Nauseef, W. M. & Borregaard, N. *Nat. Immunol.* **15**, 602–611 (2014). 10.1038/ni.2921 24940954

[3] Gong, Z. et al. *Sci. Immunol.* **8**, eadd5204 (2023). 10.1126/sciimmunol.add5204 PMC10067025 36800412

[4] Maas, R. R. et al. *Cell* **186**, 4546–4566.e27 (2023). 10.1016/j.cell.2023.08.043 37769657

[5] Ng, M. S. F. et al. *Science* **383**, eadf6493 (2024). 10.1126/science.adf6493 PMC11087151 38207030

[6] Salcher, S. et al. *Cancer Cell* **40**, 1503–1520.e8 (2022). 10.1016/j.ccell.2022.10.008 PMC9767679 36368318

[7] Xue, R. et al. *Nature* **612**, 141–147 (2022). 10.1038/s41586-022-05400-x 36352227

[8] Zhao, J. et al. *Cell Rep.* **43**, 115014 (2024). 10.1016/j.celrep.2024.115014 39630582

[9] Lad, M. et al. *Cancer Cell* **42**, 1549–1569.e16 (2024). 10.1016/j.ccell.2024.08.008 PMC11446475 39255776

[10] Wu, Y. et al. *Cell* **187**, 1422–1439.e24 (2024).

[11] Cui, A. et al. *Nature* **625**, 377–384 (2024). 10.1038/s41586-023-06816-9 PMC10781646 38057668

[12] Baessler, A. & Vignali, D. A. A. *Annu. Rev. Immunol.* **42**, 179–206 (2024). 10.1146/annurev-immunol-090222-110914 38166256

