# Supplementary material for: Common signatures of neutrophils in diverse disease conditions
Source: Cell Discov. 2025 Aug 1;11:66. doi: 10.1038/s41421-025-00818-9 (PMC12313967; doi:10.1038/s41421-025-00818-9)
Supplement: Supplementary file 1 — Supplementary information [file 41421_2025_818_MOESM1_ESM.pdf]

Supplementary Materials and Methods

Mouse information

The experimental procedures were performed in compliance with the protocols approved by the Institutional Animal Care and Use Committee (IACUC) of the Chinese Institute for Brain Research or Peking University. Mice were maintained on a 12 hr/12 hr light/dark cycle (light period 7:00 am ~ 7:00 pm) at ambient temperature (21°C ~ 24°C) with water and standard chow diet available *ad libitum* unless otherwise specified.

C57BL/6 wild-type, BALB/c wild-type, and BALB/c nude mice were purchased from Charles River International. NSG (#005557) and OT-1 (#003831) were from the Jackson Laboratory. *Ifngr1*$^{-/-}$ mice (#NM-KO-190432) on the C57BL/6 background were from Shanghai Model Organisms Center. *Rag2*$^{-/-}$ mice (#C001324) and *Rag2*$^{-/-}$ *Il2rg*$^{-/-}$ mice (#C001367) on the C57BL/6 background were from Cyagen Bioscience. 8- to 12-week-old female mice were utilized in the experiments unless otherwise specified.

Mouse disease models

For the model of metabolic disorder, 5-week-old C57BL/6 male mice were fed with a high-fat diet containing 60% of calories from fat, 20% of calories from carbohydrates, and 20% of calories from proteins (Research Diets) for 24 weeks.

For the model of experimental autoimmune encephalomyelitis (EAE), myelin oligodendrocyte glycoprotein peptide (MOG_{35-55}; Sangon Biotech) was dissolved in phosphate-buffered saline (PBS) and emulsified with an equal volume of complete Freund's adjuvant (Chondrex) to achieve a final concentration of 1 mg/ml. The antigen

emulsion was subcutaneously injected at four sites (50 μl per site) on the back of each mouse weekly for three weeks. Pertussis toxin (Sigma; 200 ng per mouse) was intraperitoneally administered immediately after and 48 hr after the first immunization. Tissues were harvested 45 days after the first MOG_{35-55} immunization.

For the model of acute lung injury (ALI), mice were briefly anesthetized with 3% isoflurane. 80 μg of lipopolysaccharide (LPS; Sigma) dissolved in 40 μl of sterile PBS was intranasally administered to each C57BL/6 female mouse. Tissues were harvested 48 hr after the LPS challenge. For the administration of the anti-Cd244 neutralizing antibody (clone OX-122, Biorbyt), each mouse was intraperitoneally injected with 20 μg of antibody immediately after the LPS challenge.

For the model of Lewis lung carcinoma (LLC), 2×10^6 LLC cancer cells suspended in 200 μl Leibovitz's L-15 medium (Thermo Fisher Scientific) were inoculated into each mouse via intravenous injection. Tissues were harvested 30 days after the cancer cell inoculation.

For the model of B16 melanoma or MC38 colorectal carcinoma, 1×10^6 B16-OVA or MC38 cancer cells suspended in 100 μl Matrigel (Corning) were subcutaneously injected at the right flank of each mouse. Tissues were harvested 21 days after the cancer cell inoculation.

For the model of intracranial glioma tumors, mice were anesthetized and head-fixed on the stereotactic frame. The head skin was shaved and prepared with iodine and 75% alcohol. A skin incision was made along the midline to expose the skull. The following coordinates for injection were measured relative to the bregma: anteroposterior = 0.5 mm, medial-lateral = 1.9 mm, and dorsal-ventral = -3.2 mm. A small hole was

drilled through the skull, through which a Hamilton syringe needle (701N, Hamilton) was inserted into the brain. 5×10^4 LCPNS or LCPNS-SIIN cancer cells were suspended in 5 µl Matrigel and delivered at 1.5 µl/min. The needle was kept in place for an additional 5 min to prevent the leakage of injected cells. Bone wax was used to cover the small hole in the skull, and the head skin was sutured. Tissues were harvested 21 days after the cancer cell inoculation.

Cancer cell cultures

LCPNS or LCPNS-SIIN glioma cell lines were established as we recently reported [1]. LCPNS or LCPNS-SIIN cells were cultured at 37°C under 5% CO_2 / 5% O_2 in Dulbecco's Modified Eagle Medium/Nutrient Mixture F-12 (DMEM/F12 medium; Gibco) supplemented with 1% N2 (Gibco), 1% B-27 (Gibco), 1% GlutaMAX (Gibco), 100 U/ml penicillin, 100 µg/ml streptomycin, 16.6 mM D-glucose, 5 mM HEPES, 50 nM 2-mercaptoethanol (Sigma), 20 ng/ml EGF (Novoprotein), and 20 ng/ml FGF2 (Origene).

LLC, B16-F10, and MC38 cell lines were purchased from the Chinese National Infrastructure of Cell Line Resource and tested negative for mycoplasma. B16-OVA cancer cells were derived by transducing with a lentivirus expressing full-length ovalbumin cDNA. LLC, B16-OVA, and MC38 cells were cultured at 37°C under 5% CO_2 in Dulbecco's Modified Eagle Medium (DMEM; Gibco) supplemented with 10% fetal bovine serum (FBS; VISTECH), 100 U/mL penicillin, and 100 µg/mL streptomycin.

Fluorescence-activated cell sorting (FACS)

For preventing the potential *ex vivo* activation of neutrophils [2,3], FACS procedures

were completed within 6 hr and on ice or at 4°C whenever possible to minimize the impact on neutrophil transcriptional states. Notably, recent published scRNA-seq studies of neutrophils have adopted similar procedures [1,4-6].

The peripheral blood of each mouse was collected via retro-orbital bleeding into PBS containing 5 mM Na-EDTA (pH 8.0).

The spleen of each mouse was freshly dissected and directly mashed through a 70-μm cell strainer in Hank's Balanced Salt Solution (HBSS) containing 3% heat-inactivated fetal bovine serum (HI-FBS; Sigma).

The tibias of each mouse were freshly dissected, and the bone marrow was flushed out with HBSS containing 3% HI-FBS. The skulls of each mouse were freshly dissected, and the attached dura mater was removed. The skulls were cut into small pieces and incubated with 360° rotation in RPMI-1640 medium (Gibco) containing 3% HI-FBS and 10 mM HEPES at 37°C for 10 min to flesh out the bone marrow. Cell suspensions of the bone marrow were filtered through 70-μm cell strainers.

The spinal cord of each mouse was freshly dissected and cut into small pieces on ice. The tissue was digested in an adequate volume of Accutase (BioLegend) at 37°C for 15 min and then mashed through a 70-μm cell strainer.

The lungs of each mouse were freshly dissected and cut into small pieces on ice. The tissue was digested in RPMI-1640 medium containing 0.1 mg/ml Liberase TL (Roche), 20 μg/ml DNase I (Sigma), 10 mM HEPES, and 3% HI-FBS at 37°C for 15 min and then mashed through a 70-μm cell strainer.

LLC, B16, or MC38 peripheral tumors were freshly dissected and cut into small pieces on ice. The tumor tissues were digested in RPMI-1640 medium containing 0.1

mg/ml Liberase TL, 20 μg/ml DNase I, 10 mM HEPES, and 3% HI-FBS at 37°C for 15 min and then mashed through 70-μm cell strainers.

LCPNS or LCPNS-SIIN glioma tumors were freshly dissected and cut into small pieces on ice. The tumor tissues were digested in an adequate volume of Accutase at 37°C for 15 min and then mashed through 70-μm cell strainers.

Cell suspensions prepared from different tissues were centrifuged at 500 *g* for 5 min. The cells were resuspended in ammonium-chloride-potassium (ACK; Thermo Fisher Scientific) to lyse red blood cells. The cells were centrifuged again at 500 *g* for 5 min and resuspended in HBSS containing 10 mM EDTA-Na (pH 8.0) and 2% HI-FBS for staining with the intended FACS antibodies and 7-AAD (Thermo Fisher Scientific). FACS antibodies utilized in this study included Cd45-PE (#103106, BioLegend), Cd45-PE-Cy7 (#103114, BioLegend), Cd11b-BV510 (#101263, BioLegend), Ly6G-BV421 (#127628, BioLegend), Ly6G-FITC (#127606, BioLegend), Ly6C-APC-Cy7 (#128026, BioLegend), Cd274-APC (#124312, BioLegend), MHCII-PE (#107607, BioLegend), Cd101-AF700 (#56-1011-82, eBioscience), Il4ra-AF488 (#53-1241-82, eBioscience), and Cd244a-PE-Cy7 (#133511, BioLegend).

The stained cells were processed on the BD LSRFortessa, and the data were analyzed by FlowJo (https://www.flowjo.com). Alternatively, neutrophils ($Cd45^+$ $Cd11b^+$ $Ly6G^+$ $Ly6C^{low}$) were sorted on BD FACSAria.

Single-cell RNA sequencing (scRNA-seq)

Singleron Matrix Workflow: Single-cell suspensions (2×10^5 cells/ml) in PBS were prepared and loaded onto a microwell chip using the Singleron Matrix Single Cell

Processing System. Barcoding beads were collected from the chip, and mRNAs captured on beads underwent reverse transcription. The resulting cDNAs were amplified by PCR, fragmented, and ligated with sequencing adapters. Single-cell RNA-seq libraries were constructed following the manufacturer's protocol for the GEXSCOPE Single Cell RNA Library Kit. The libraries were diluted to 4 nM and sequenced on the Illumina NovaSeq 6000 platform using 150 base pair (bp) paired-end reads.

SeekOne Workflow: Single-cell RNA-seq libraries were generated using the SeekOne Digital Droplet Single Cell 3' Library Preparation Kit (SeekGene). Single-cell suspensions were mixed with reverse transcription reagents and loaded into the sample wells of the SeekOne Chip S3. Barcoded hydrogel beads and partitioning oil were dispensed separately to generate droplets containing single cells. Reverse transcription was performed within the emulsion droplets, followed by droplet breaking to release cDNAs, which were subsequently purified and amplified. The amplified cDNAs were then processed through fragmentation, end-repair, A-tailing, and ligation to sequencing adapters. An indexed PCR was performed to amplify DNA corresponding to the 3' polyA tails of genes, incorporating both the Cell Barcode and the Unique Molecular Index (UMI). The indexed libraries were purified using VAHTS DNA Clean Beads (Vazyme) and assessed for quality and concentration using the Qubit (Thermo Fisher Scientific) and the Bio-Fragment Analyzer (BiOptic). The libraries were sequenced on the Illumina NovaSeq X Plus platform with 150 bp paired-end reads.

scRNA-seq data processing

Data preprocessing was performed according to the manufacturer's reference

instructions. The reference mouse genome used for sequence alignment was mm10. For quality control, we filtered out genes expressed in <10 cells, as well as cells expressing gene numbers <200, mitochondrial reads >10%, or ribosome genes >20%. We used the Solo in scvi-tools to remove doublets using the top 2,000 variant genes and default parameters.

To integrate scRNA-seq data, we adopted the methods of recent scRNA-seq studies of neutrophils [1,4-6]. We utilized single-cell Variational Inference (scVI) (https://docs.scvi-tools.org/en/stable/tutorials/) for data integration [7]. Data clustering was performed using Scanpy (https://github.com/scverse/scanpy), and the resulting H5ad files were converted to Seurat objects using Sceasy (https://github.com/cellgeni/sceasy). The analysis was conducted in RStudio with R v4.2.2 (https://cran.r-project.org/) and Seurat v4 (https://satijalab.org/seurat/articles/get_started.html). Marker genes for each cluster were identified and visualized using the FindAllMarkers function in Seurat. Cell type annotation was initially performed using a combination of ScType (https://sctype.app) and PanglaoDB (https://panglaodb.se/index.html).

For detailed characterization of neutrophil transcriptional states, we referenced previously published studies [1,8,9]. To ensure balanced visualization across samples, we down-sampled each sample to a maximum of 5,000 cells during the UMAP embedding stratified by tissue types or disease conditions.

Analyses of RNA velocity, pseudotime, and gene regulation

RNA velocity analysis was performed using scVelo (https://scvelo.readthedocs.io/en/stable/) with default parameters. The input data for

scVelo was generated using the Python implementation of Velocyto.

Pseudotime trajectory analysis was conducted with Monocle 3 (https://cole-trapnell-lab.github.io/monocle3/) with default settings.

We employed pySCENIC (v0.12.0) in a Docker environment (https://hub.docker.com/r/aertslab/pyscenic) to investigate gene regulatory networks. Differentially-expressed transcription factors were identified based on AUC scores and visualized by ComplexHeatmap (https://www.bioconductor.org/packages/devel/bioc/html/ComplexHeatmap.html) and ClusterProfiler (https://bioconductor.org/packages/release/bioc/html/clusterProfiler.html).

Gene set enrichment analysis was conducted using the Python version of DecoupleR (v1.7.1) (https://github.com/saezlab/decoupler-py) in combination with Scanpy (v1.10.2).

The Over Representation Analysis (ORA) method was applied to gene sets from MSigDB (v7.4) (https://www.gsea-msigdb.org/gsea/msigdb), with a focus on cytokine-associated gene sets.

Mapping cytokine datasets to neutrophil subtypes

Raw FASTQ files containing single-cell transcriptomic profiles of immune cells exposed to various cytokines were obtained from the Gene Expression Omnibus (GEO) database under accession number GSE202186 [10]. Sequencing reads were aligned to the reference mouse genome mm10, and transcriptomic count matrices were generated using the CellRanger pipeline (v7.2). For the hashtag library, FASTQ files were processed with CITE-seq-Count (v1.4.3) (github.com/Hoohm/CITE-seq-Count). Gene expression data

were matched with hashtag information using the HTODemux function in the Seurat R package (v5.1.0) (https://satijalab.org/seurat/). Cells classified as multiplets (e.g., those with multiple hashtags) were excluded from downstream analyses.

Quality control was performed using the Scanpy package (v1.10.3). Cells were retained if they expressed <6,000 genes and mitochondrial gene contents <20%. Gene expression matrices were normalized by scaling each cell's gene expression to its total expression, multiplying by a scaling factor of 10,000, and applying a log transformation. For dimensionality reduction, the top 2,000 variable genes were selected. Principal component analysis (PCA) was then used to denoise the data and reduce it to a lower-dimensional representation, retaining the top 40 principal components that were used for global clustering and visualization with UMAP.

Neutrophils were identified at the cluster level based on UMAP clustering results and the expression of canonical marker genes, including *S100a8*, *Cxcr2*, *Itgam*, *Mmp9*, and *Csf3r*. Cytokine information for each neutrophil was inferred from the corresponding hashtags. Finally, the Seurat R package was used to map neutrophil clusters to neutrophil transcriptional states defined in the current study, enabling the identification of cytokines potentially regulating each state.

Bulk RNA sequencing (RNA-seq)

Total RNAs of neutrophils were extracted and reverse-transcribed using a template-switching oligo (TSO; BGI Genomics). cDNAs underwent pre-amplification and tagmentation to add sequencing adapters, and the index PCR was performed to incorporate sample-specific barcodes. The libraries were purified using VAHTS DNA

Clean Beads (Vazyme) and assessed for quality and concentration using the Qubit (Thermo Fisher Scientific) and the Bio-Fragment Analyzer (BiOptic). The libraries were sequenced on the MGI DNBSEQ-T7 platform using 150 bp paired-end reads.

Sequencing files were aligned to the reference mouse genome mm10 using STAR (v2.7.11a) (https://code.google.com/archive/p/rna-star/). Gene expression levels were quantified with FeatureCounts included in the Subread package (https://subread.sourceforge.net/). Differential gene expression analysis was performed using edgeR (https://bioconductor.org/packages/release/bioc/html/edgeR.html). Pathway enrichment analysis was conducted using the fgsea package (https://bioconductor.org/packages/release/bioc/html/fgsea.html), with results visualized through ggplot2 (https://ggplot2.tidyverse.org) for the representation of enriched pathways from MSigDB (v7.4).

***In vitro* treatments**

Neutrophils were FACS-sorted from the tibial bone marrow of C57BL/6 wild-type mice and cultured in RPMI-1640 medium supplemented with 10% FBS, 100 U/mL penicillin, and 100 μg/mL streptomycin in 6-well plates (1×10^6 neutrophils per well). Neutrophils were *in vitro* treated with a final concentration of 20 ng/ml IL-6 (PeproTech), TGF-β (Novoprotein), TNF-α (Novoprotein), IFN-α (Sinobiological), IFN-β (Sinobiological), or IFN-γ (Novoprotein) for 18 hr. Neutrophils were then examined by FACS or bulk RNA-seq analyses.

The co-cultures of neutrophils and $Cd8^+$ T cells were performed as we recently reported [1]. The spleen and lymph nodes of OT-1 mice were freshly dissected and mashed

through a 70-μm cell strainer in HBSS containing 3% HI-FBS. The cells were centrifuged at 500 *g* for 5 min and resuspended in ACK buffer to lyse red blood cells. The cells were centrifuged again at 500 *g* for 5 min and resuspended in RPMI 1640 medium supplemented with 10% HI-FBS, 2 mM glutamine, 55 μM 2-mercaptoethanol, 1 mM sodium pyruvate, 100 U/ml penicillin, and 100 μg/ml streptomycin. After loading with 1 mM $OVA_{257-264}$ peptide (SIINFEKL) at 37°C for 1 hr, the cells were centrifuged at 500 *g* for 5 min, resuspended in the culture medium, and cultured at 37°C overnight. The activated OT-1 $Cd8^+$ T cells ($Cd45^+ Cd3^+ Cd4^- Cd8^+ NK1.1^-$) were then FACS sorted. In parallel, $Cd244^+$ neutrophils from the peripheral blood or $Cd274^+$ neutrophils from the lungs of C57BL/6 wild-type mice in the ALI model were FACS sorted. 5×10^4 OT-1 $Cd8^+$ T cells were co-cultured with 5×10^4 neutrophils for 6 hr before the expression of specific exhaustion markers was analyzed by FACS.

Data availability

The sequencing data generated in this study are publicly available without restriction. scRNA-seq and bulk RNA-seq FASTQ files have been deposited in the Genome Sequence Archive at the National Genomics Data Center under the accession number CRA020963 (https://ngdc.cncb.ac.cn/bioproject/browse/CRA020963). The fully processed single-cell expression matrix, together with cell-level metadata, UMAP coordinates, and gene annotations, is provided as an h5ad file (Scanpy-compatible) from Figshare (https://doi.org/10.6084/m9.figshare.29538791).

Statistical analyses

Statistical analyses were performed by GraphPad Prism 9.5.0 (http://www.graphpad.com/scientific-software/prism). All the sample points (n) represent biological replicates (i.e., mice or cell preparations). The statistical test description and *p*-values are included in the figure legends where appropriate.

References

1 Zhao, J. *et al.* Disease-specific suppressive granulocytes participate in glioma progression. *Cell Rep* **43**, 115014, doi:10.1016/j.celrep.2024.115014 (2024).

2 Oh, H., Siano, B. & Diamond, S. Neutrophil isolation protocol. *J Vis Exp*, doi:10.3791/745 (2008).

3 Li, C. *et al.* The effect of whole blood logistics on neutrophil non-specific activation and kinetics ex vivo. *Sci Rep* **14**, 2543, doi:10.1038/s41598-023-50813-x (2024).

4 Xie, X. *et al.* Single-cell transcriptome profiling reveals neutrophil heterogeneity in homeostasis and infection. *Nat Immunol* **21**, 1119-1133, doi:10.1038/s41590-020-0736-z (2020).

5 Grieshaber-Bouyer, R. *et al.* The neutrotime transcriptional signature defines a single continuum of neutrophils across biological compartments. *Nat Commun* **12**, 2856, doi:10.1038/s41467-021-22973-9 (2021).

6 Salcher, S. *et al.* High-resolution single-cell atlas reveals diversity and plasticity of tissue-resident neutrophils in non-small cell lung cancer. *Cancer Cell* **40**, 1503-1520 e1508, doi:10.1016/j.ccell.2022.10.008 (2022).

7 Luecken, M. D. *et al.* Benchmarking atlas-level data integration in single-cell

genomics. *Nat Methods* **19**, 41-50, doi:10.1038/s41592-021-01336-8 (2022).

8 Ng, M. S. F. *et al.* Deterministic reprogramming of neutrophils within tumors. *Science* **383**, eadf6493, doi:10.1126/science.adf6493 (2024).

9 Wu, Y. *et al.* Neutrophil profiling illuminates anti-tumor antigen-presenting potency. *Cell* **187**, 1422-1439 e1424, doi:10.1016/j.cell.2024.02.005 (2024).

10 Cui, A. *et al.* Dictionary of immune responses to cytokines at single-cell resolution. *Nature* **625**, 377-384, doi:10.1038/s41586-023-06816-9 (2024).

Supplementary Table

Supplementary Table S1. Mouse information for scRNA-seq analyses of neutrophils

Disease Model	Mouse Strain	Sex	Age at Tissue Collection	Numbers (n)
Control condition	C57BL/6 wild-type	Female	12 weeks	10
High-fat diet	C57BL/6 wild-type	Male	29 weeks	5
Experimental autoimmune encephalomyelitis	C57BL/6 wild-type	Female	15 weeks	10
Acute lung injury	C57BL/6 wild-type	Female	12 weeks	5
Lewis lung carcinoma	C57BL/6 wild-type	Female	12 weeks	5
B16 melanoma	C57BL/6 wild-type	Female	11 weeks	15
MC38 colorectal carcinoma	C57BL/6 wild-type	Female	11 weeks	15
LCPNS-SIIN glioma	C57BL/6 wild-type	Female	11 weeks	15
LCPNS glioma	C57BL/6 wild-type	Female	11 weeks	15
LCPNS-SIIN glioma in NSG	NSG	Female	11 weeks	15

Supplementary Figures

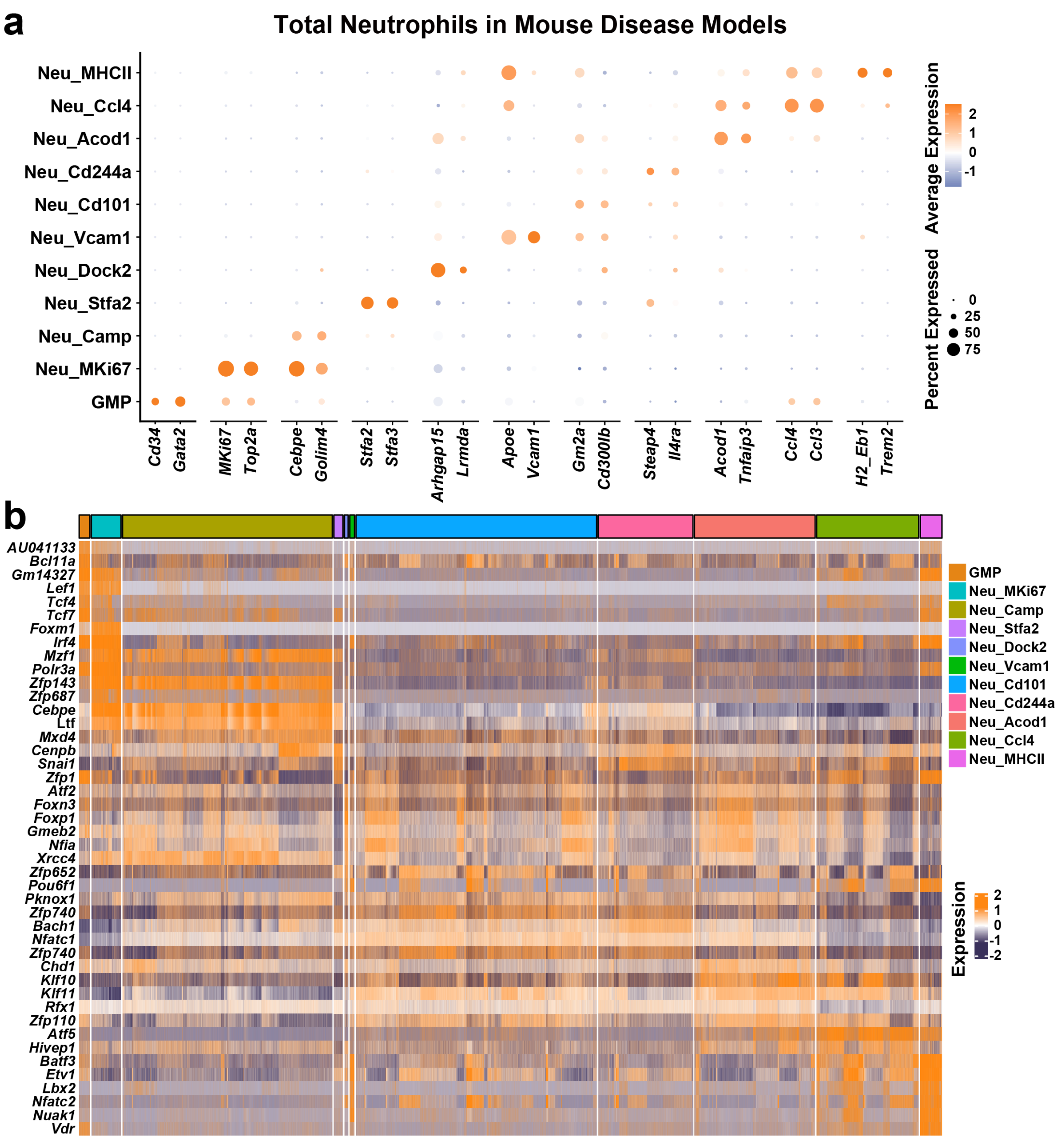

Supplementary Figure S1. Signature genes of neutrophil transcriptional states.

a Dot plot of the two top marker genes for each neutrophil transcriptional state defined in the pooled scRNA-seq dataset of mouse disease models.

b Heatmap of SCENIC binary regulon activities in neutrophil transcriptional states.

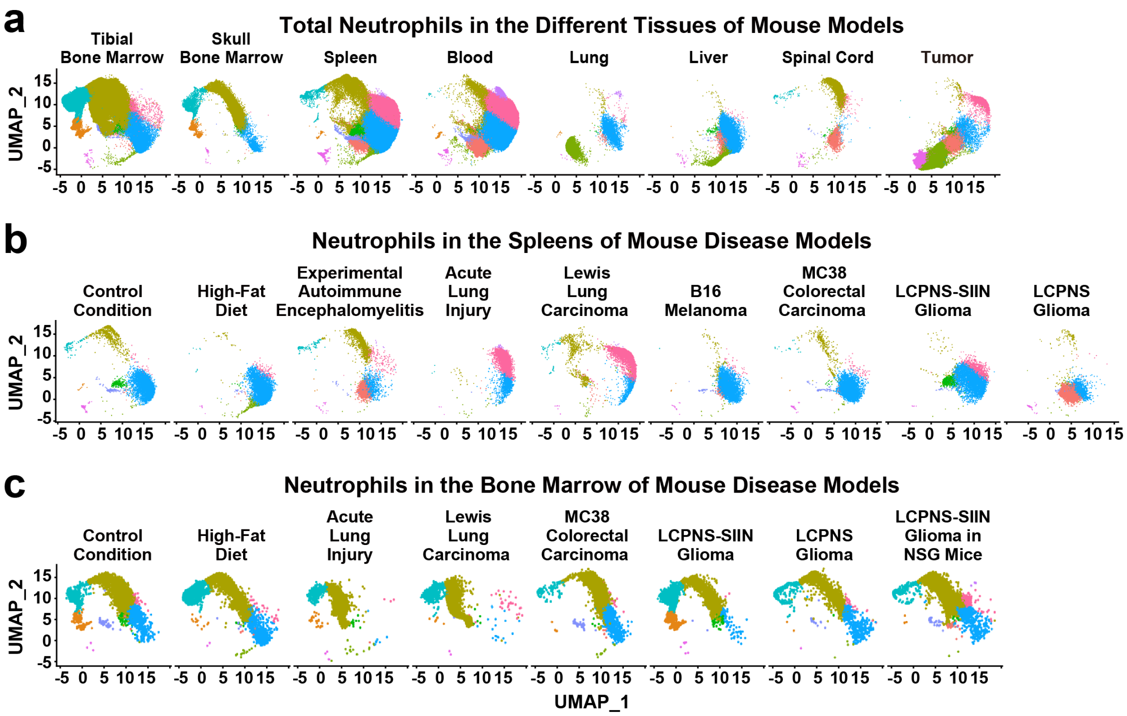

Supplementary Figure S2. Neutrophil transcriptional states in different tissues and disease conditions.

a UMAP plots of neutrophil transcriptional states in different tissues defined in the pooled scRNA-seq dataset of mouse disease models.

b, c UMAP plots of neutrophil transcriptional states in the spleen **(b)** or bone marrow **(c)** of mouse disease models.

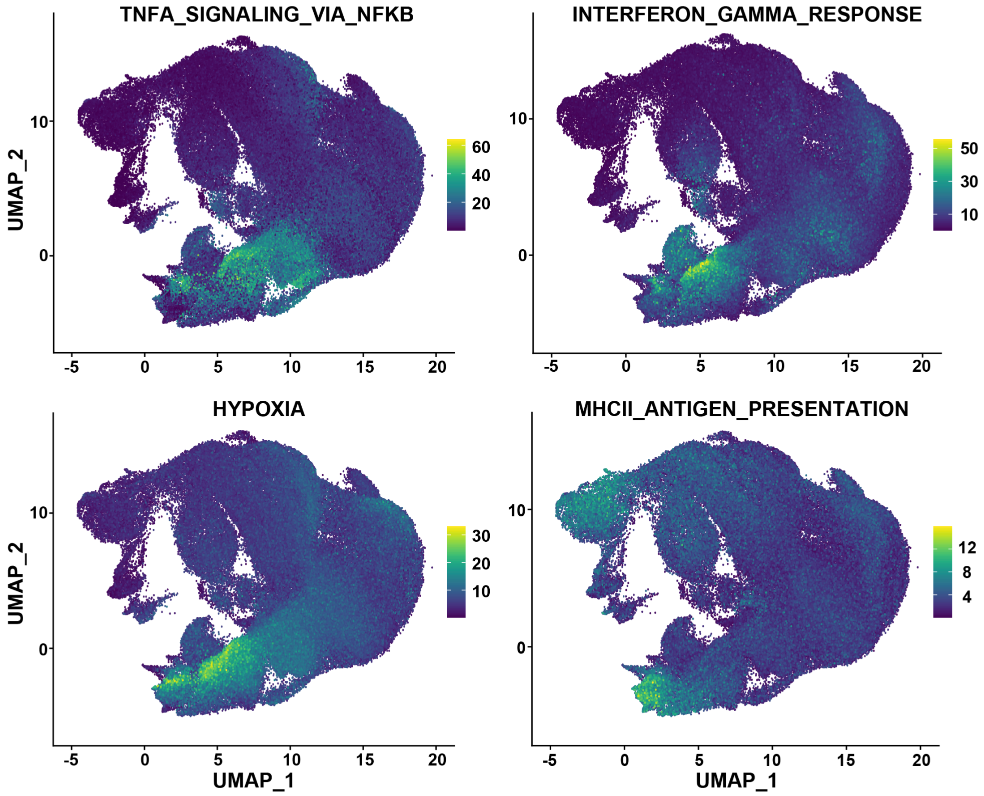

Supplementary Figure S3. Enrichment of signaling pathways in neutrophil transcriptional states.

Enrichment scores for the gene sets of specific signaling pathways calculated by the Over Representation Analysis are projected onto the UMAP plots of neutrophil transcriptional states defined in the pooled scRNA-seq dataset of mouse disease models.

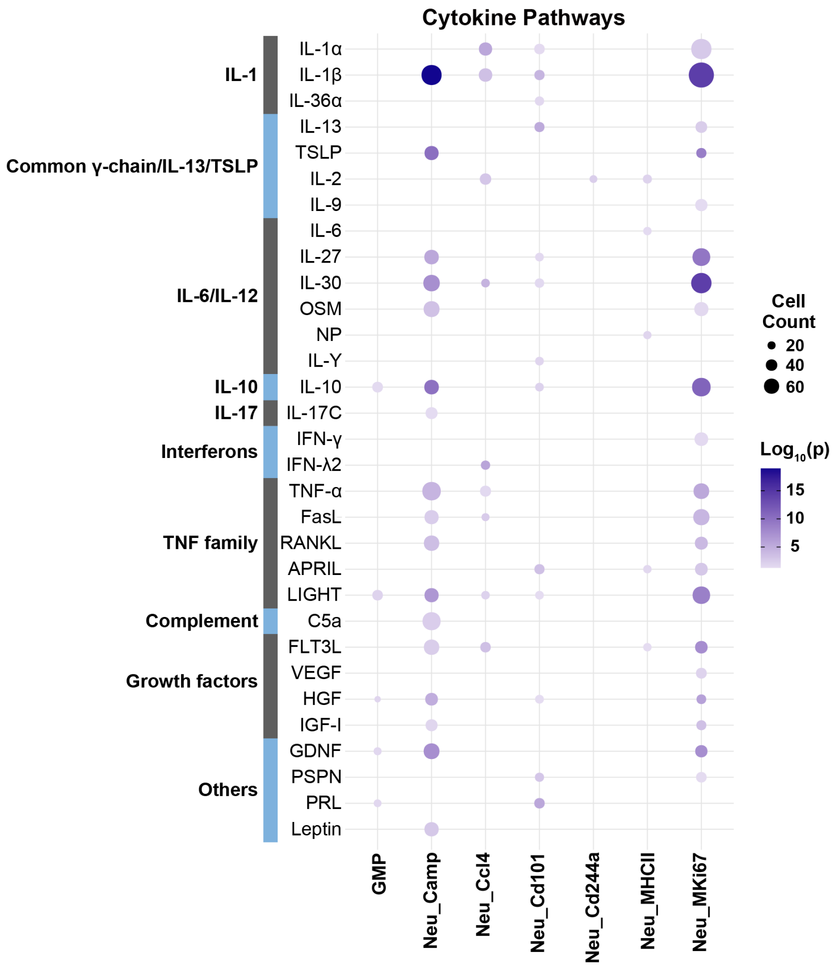

Supplementary Figure S4. Cytokine signals for neutrophil transcriptional states.

The published scRNA-seq dataset of immune cells exposed to various cytokines (GSE202186) was re-analyzed. Neutrophils within this dataset were mapped to transcriptional states defined in the current study.

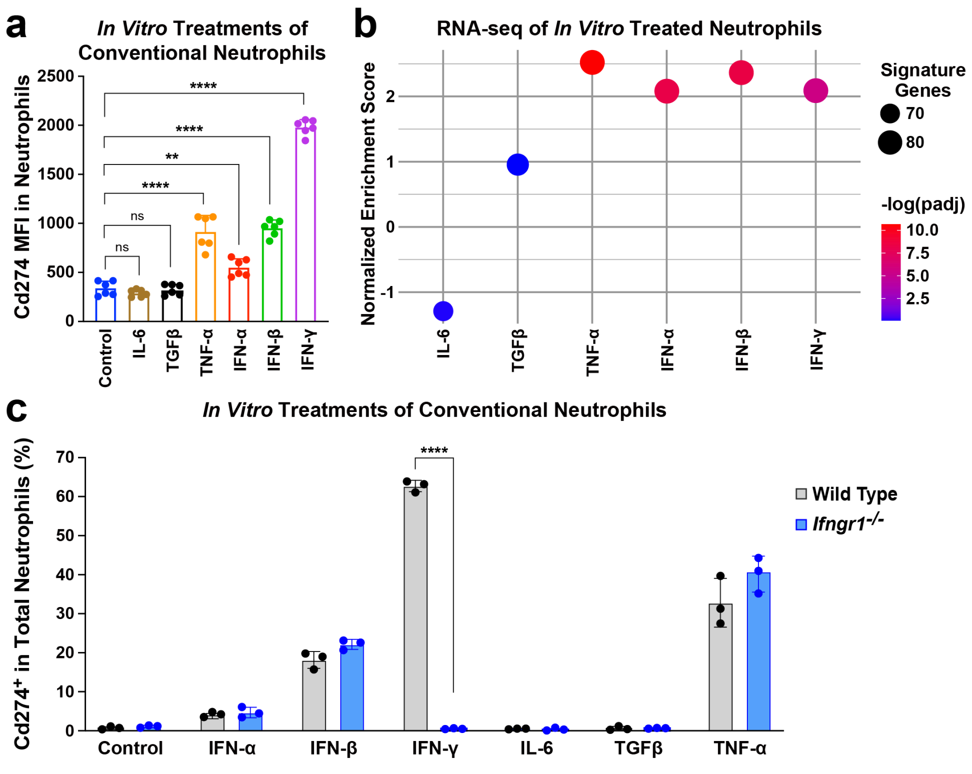

Supplementary Figure S5. *In vitro* induction of Neu_Ccl4 by specific cytokines.

a, b Neutrophils were FACS-sorted from the tibial bone marrow of C57BL/6 wild-type mice and *in vitro* treated with the indicated cytokines. **b** Mean fluorescence intensity (MFI) of Cd274 expression in neutrophils was determined by FACS. Mean ± SD, ns not significant, ** $p < 0.01$, **** $p < 0.0001$ (ANOVA test). **b** Neutrophils treated with the indicated cytokines were profiled by bulk RNA-seq. Normalized enrichment scores of the top 100 marker genes of Neu_Ccl4 are shown.

c Neutrophils were FACS-sorted from the tibial bone marrow of *Ifngr1*$^{-/-}$ mice and *in vitro* treated with the indicated cytokines. Cd274^{+} neutrophils were quantified by FACS. Mean ± SD, **** $p < 0.0001$ (Student's *t*-test).

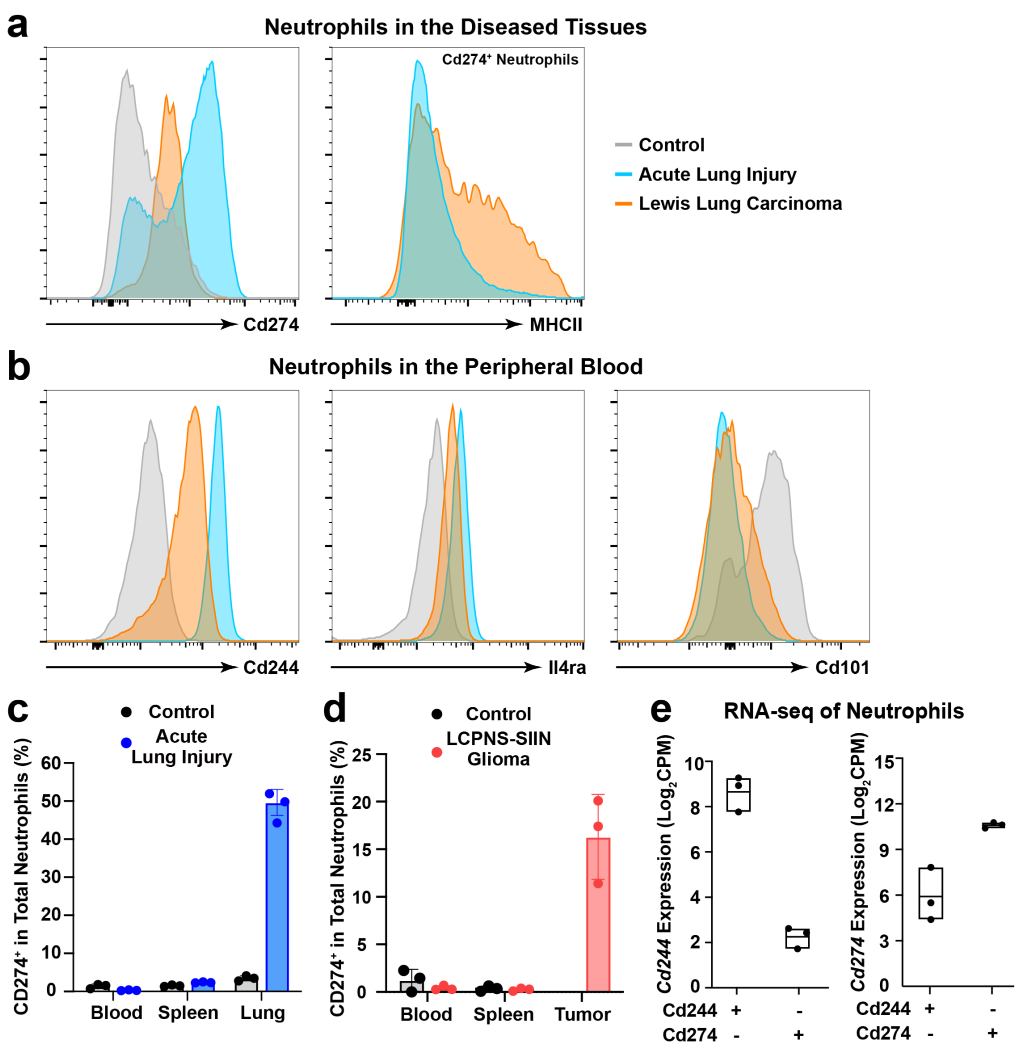

Supplementary Figure S6. Characterization of neutrophil transcriptional states.

C57BL/6 wild-type mice were subjected to the indicated disease models.

a Cd274 and MHCII expression by neutrophils in the lungs of control mice, the lungs of the ALI model, or the lung tumors of the LLC model were examined by FACS. Representative histograms are shown.

b Cd244, Il4ra, and Cd101 expression by neutrophils in the peripheral blood of control mice, the ALI model, or the LLC model was assessed by FACS. Representative histograms are shown.

c Cd274$^+$ neutrophils in the peripheral blood, spleen, and lungs of control mice or the ALI model were quantified by FACS. Mean ± SD.

d Cd274$^+$ neutrophils in the peripheral blood, spleen, and intracranial tumors of control mice or the LCPNS-SIIN glioma model were determined by FACS. Mean ± SD.

e Cd244$^+$ neutrophils from the peripheral blood and Cd274$^+$ neutrophils from the lungs of the ALI model were profiled by bulk RNA-seq. Expression levels of *Cd244* and *Cd274* are presented as box plots.

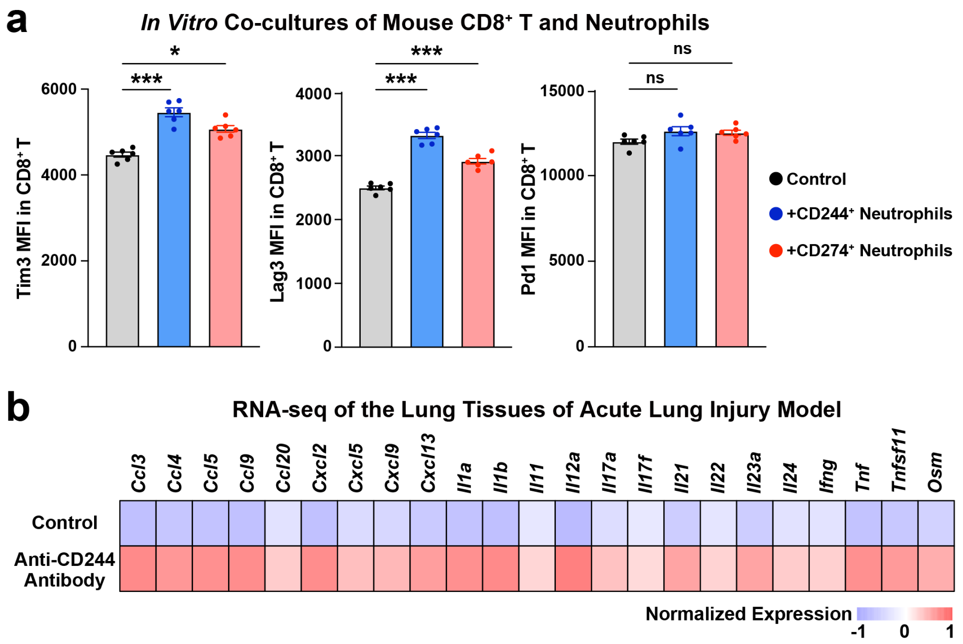

Supplementary Figure S7. Immunosuppressive function of Cd244$^+$ neutrophils.

a Cd244$^+$ neutrophils from the peripheral blood or Cd274$^+$ neutrophils from the lungs of C57BL/6 wild-type mice in the ALI model were *in vitro* co-cultured with OT-1 Cd8$^+$ T cells. Mean fluorescence intensity (MFI) of Tim3, Lag3, or Pd1 expression by Cd8$^+$ T cells was determined by FACS. Mean ± SEM, ns not significant, * $p < 0.05$, *** $p < 0.001$ (ANOVA test).

b C57BL/6 wild-type mice were treated with an anti-Cd244 neutralizing antibody immediately after the ALI model. The lung tissues were profiled by bulk RNA-seq (five mice per condition). Average expression of pro-inflammatory cytokines and chemokines up-regulated in the anti-Cd244 condition are shown.
